# Functional Dyspepsia, Peptic Ulcer, and *Helicobacter pylori* Infection in a Rural Community of South Asia: An Endoscopy-Assisted Household Survey

**DOI:** 10.14309/ctg.0000000000000334

**Published:** 2021-04-16

**Authors:** M. Masudur Rahman, Uday C. Ghoshal, Md. Golam Kibria, Nigar Sultana, M. Abdllah Yusuf, Shamsun Nahar, Faruque Ahmed, AHM Rowshon, Mahmud Hasan

**Affiliations:** 1 Department of Gastroenterology, Sheikh Russel National Gastroliver Institute and Hospital, Dhaka, Bangladesh;; 2 Department of Gastroenterology, Sanjay Gandhi Postgraduate Institute of Medical Sciences, Lucknow, India;; 3 Department of Gastroenterology, Delta Medical College and Hospital, Dhaka, Bangladesh;; 4 Department of Microbiology, National Institute of Neuroscience and Hospital, Dhaka, Bangladesh;; 5 Laboratory Science and Service Division, icddr,b, Dhaka, Bangladesh;; 6 Department of Gastroenterology, Shaheed Suhrawardy Medical College, Dhaka, Bangladesh;; 7 Gastroliver Foundation, Dhaka, Bangladesh.

## Abstract

Functional dyspepsia (FD), although commoner than organic dyspepsia (OD) in-hospital studies, community data, particularly from rural areas, are lacking. We performed a rural community study in Bangladesh with the primary aims to evaluate (i) the prevalence of uninvestigated dyspepsia (UD), FD, and OD and (ii) the risk factors for UD.

**METHODS:**

This house-to-house survey was performed using a translated-validated enhanced Asian Rome III questionnaire and endoscopy with *Helicobacter pylori* tests, including genotyping.

**RESULTS:**

Of 3,351/3,559 responders ([94.15%], age 40.41 ± 16.05 years, female 1924 [57.4%]), 547 (16.3%) had UD (female 346 [18%] vs male 201 [14%]; *P* = 0.002); 201 (6%), 88 (2.6%), and 258 (7.7%) had postprandial distress (PDS), epigastric pain syndromes (EPS) and PDS-EPS overlap, respectively. On multivariate analysis, age >50 years (adjusted odds ratio [AOR] 1.34 [1.07–1.68]), female sex (AOR 1.42 [1.17–1.74]), being married (AOR 1.57 [1.21–2.07]), lower family income (AOR 1.79 [1.43–2.26]), nonsteroidal anti-inflammatory drug use (AOR 7.05 [2.11–23.55]), previous acute gastroenteritis (AOR 5.42 [1.83–16]), and psychological distress (AOR 5.02 [2.87–8.76]) were risk factors for UD. Of 346/547 (63.25%) undergoing endoscopy, 232 (67.05%) and 114 (32.95%) had FD and OD (peptic ulcers [PU] 99 [28.61%] and erosive esophagitis 13 [3.76%]). About 53% of FD subjects had EPS-PDS overlap, 32% had PDS, and only 15% had EPS. *H. pylori* was detected in 266/342 (78%) dyspeptics (FD 173/230 [75.2%], vs OD 92/114 [82.1%], *P* = 0.169).

**DISCUSSION:**

Sixteen percent, 11% and 5% of rural Bangladeshi Asian adults had UD, FD, and PU, respectively. One-third of UD subjects had OD, mostly PU.
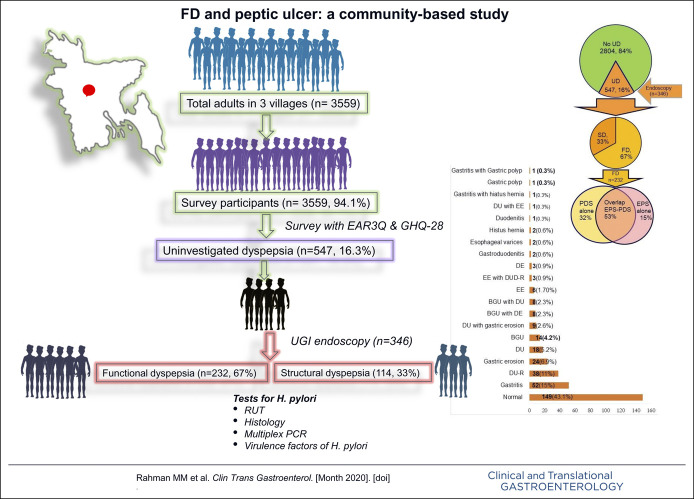

## INTRODUCTION

Dyspepsia is a common problem that causes substantial resource utilization and impairment in quality of life (QoL) (1,2). Globally, the prevalence of uninvestigated dyspepsia (UD) varies between 7% and 45% (3). The epidemiology of dyspepsia in Asia is different from the Western population (4,5). This may result from the cultural difference in symptom reporting, dietary, sociocultural, and psychological factors. Moreover, the frequency of gastrointestinal (GI) infection is common in Asia, including *Helicobacter pylori* and acute infectious gastroenteritis (AGE), which cause not only postinfection irritable bowel syndrome (IBS) but also functional dyspepsia (FD) (6,7). The prevalence of UD varies from 8% to 30% in Asia (4). Most prevalence studies are from the urban areas of developed nations and not from the rural communities of the less developed countries having a higher frequency of *H. pylori* infection and AGE (4).

Previously, epidemiological studies focused on UD rather than FD and organic dyspepsia (OD), except a few community-based studies from the West (8–10). In a meta-analysis, the frequency of FD and OD among UD subjects defined by Rome criteria in 9 studies was 82% and 18%, respectively (11). The prevalence of FD ranged from 8% to 23% in Asia (4). Most studies on dyspepsia in Asia are institution-based, suggesting that the frequency of OD, particularly *H. pylori*–associated peptic ulcers (PU) and gastric cancer, is not uncommon in Asia (12,13). However, community-based studies on FD may reflect the disease burden more accurately as most subjects with FD may not consult physicians. The community-based epidemiological studies on FD are scanty because of difficulties in excluding the organic diseases by upper gastrointestinal (UGI) endoscopy in the community (3,4). Although virulent strains of *H. pylori* among patients with FD and PU have been found comparable in hospital-based studies in high endemic areas (14), community-based data are lacking. Accordingly, we conducted a community-based endoscopy-assisted study in a rural Bangladeshi Asian population with the primary aims to evaluate (i) the prevalence of UD, FD, and OD and (ii) the risk factors for UD. The secondary aims were to evaluate (i) the prevalence of PU, (ii) the frequency of *H. pylori* in FD and PU, and (iii) the virulence-associated genes of *H. pylori* (CagA, vacA, and, specifically, vacA allelic variants) among patients with FD as compared to PU.

## METHODS

### Study design and population

**Figure 1.**
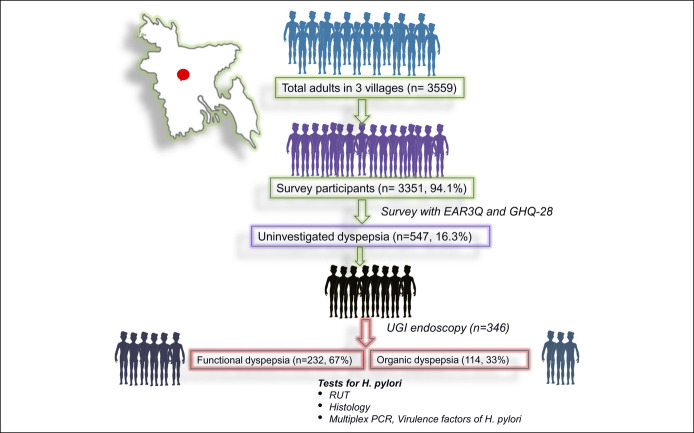
Study protocol. EAR3Q, Enhanced Asian Rome 3 questionnaire; GHQ 28, general health questionnaire-28; PCR, polymerase chain reaction; RUT, rapid urease test; UGI, upper gastrointestinal.

This prospective cross-sectional, house-to-house survey was undertaken by 3 trained interviewers during a period between November 2012 and November 2013 among the adult population (≥18 years old) in 3 villages (Charcharia, Churain of Nawabganj upazila, Dhaka district, and Kharrah of Srinagar upazila, Munshiganj district of Bangladesh) using enhanced Asian Rome III questionnaire (EAR3Q). Each subject completed the questionnaire himself/herself (except illiterate or visually impaired subjects). Dyspeptic subjects (Rome III criteria) were offered a UGI endoscopy, including *H. pylori* tests (rapid urease test [RUT], histology, and multiplex polymerase chain reaction [PCR]) from gastric biopsies (Figure 1). The protocol was approved by the Institutional Ethics Committee, and written informed consent was obtained.

### The questionnaire

The translated-validated Bengali questionnaire had subsections on sociodemographic and clinical profiles, including EAR3Q (15), and general health questionnaire-28 (GHQ-28). GHQ-28 included (i) somatic symptoms, (ii) anxiety and insomnia, (iii) social dysfunction, and (iv) depression (16). For GHQ-28 calculation, each question of the 4 domains were scored from 0 to 3; the scores of all the 28 questions were summed up to calculate the total score (≥23 considered abnormal).

### UGI endoscopy

The subjects fulfilling the Rome III dyspepsia criteria underwent UGI endoscopy (Pentax, Tokyo, Japan) by 2 experienced Gastroenterologists (MMR and MGK) in a local facility. Three biopsies each from the gastric antrum and body were obtained.

### H. pylori

RUT (urea agar-based medium), histopathology (Giemsa, and hematoxylin and eosin stains), and multiplex PCR for *H. pylori* were performed on the gastric biopsies. *H. pylori* was also genotyped by a validated multiplex PCR assay directly from the gastric biopsy (17). *H. pylori* was considered positive if either PCR or both RUT and histology were positive.

### Definitions

UD, functional heartburn (FH), and IBS were defined by the Rome III criteria (18). Dyspepsia was subclassified into epigastric pain syndromes (EPS), postprandial distress syndromes (PDS), and EPS-PDS overlap (18). Significant impairment of QoL was considered when symptoms affected it a lot or a great deal. The PU was defined as an area of the denuded epithelium of 5 mm or more. Acute gastric or duodenal erosions were defined as a mucosal break of ≤ 5 mm in diameter. All gastric ulcers were biopsied for histology. Duodenal bulb deformity was considered when flattening, scars, stenosis, or narrowing of the bulb were seen.

FD was defined as dyspepsia without any structural disease that was likely to explain symptoms. Duodenitis or duodenal erosions, PU, and erosive esophagitis (EE) defined OD. Gastritis or gastric erosion was not included in OD because these do not cause dyspeptic symptoms, do not incur the risk of PU (19), and were included as FD in a previous study (20). On the other hand, endoscopic duodenitis or duodenal erosion correlates with dyspeptic symptoms and response to treatment (20,21).

### Statistical analysis

A data-entry operator entered the data, 10% of which were cross-checked by the 2 investigators (MMR and NS). Statistical analysis was performed using SPSS version 15 (SPSS, Chicago, IL) and R Studio software (R Core Team [2013], Vienna, Austria). Data were presented as proportion, mean and standard deviation (SD) or median and interquartile range depending on the type and distribution. Normally distributed continuous data were analyzed using the unpaired *t* test. Categorical and nonparametric continuous data were analyzed using χ^2^ and Mann-Whitney *U* tests, respectively. Binary variables found significant in univariate analysis were entered into stepwise logistic regression and likelihood ratio tests for multivariate analysis. *P* values less than 0.05 were considered significant.

## RESULTS

### Demographic and socioeconomic characteristics

Of the 3,559 subjects, 3,351 (94.15%) responded (Figure 1). Of them, 1,924 (57.4%) were women. The mean age of the study subjects was 40.4 ± 16.0 years.

### Uninvestigated dyspepsia

The prevalence of UD was 16.3% (Figure 1). Table 1 shows the demographic and socioeconomic characteristics of the subjects with and without UD. The prevalence of UD was higher among women (18%) compared with men (14%) (*P* = 0.002). The number of subjects with psychological distress (GHQ-28 score ≥ 23) was higher among participants with than without UD. Somatic symptoms, anxiety, insomnia, depression score, and the total score of GHQ 28 were also higher among subjects with than without UD (Table 1).

**Table 1.** Sociodemographic and psychological characteristics of subjects with and without UD

Characteristics	Subjects with UD (n = 547)	Subjects without UD (n = 2,804)	*P* value
Age, (yrs, mean ± SD)	42.70 ± 14.43	39.96 ± 16.31	<0.001
Male sex, n (%)	201 (36.7)	1,226 (43.7)	0.002
Marital status, n (%)			0.001
Married	470 (85.4)	2,208 (79.6)	
Single	77 (14.1)	565 (20.4)	
Education, n (%)			0.017
Illiterate and up to class V	319 (58.3)	1,479 (52.7)	
Class VI and above	228 (41.7)	1,325 (47.3)	
Family income (taka/mo)^a^ mean ± SD	9,814.92 ± 6,146.38	11,625.69 ± 8,414.43	<0.001
Occupation, n (%)			<0.001
Housewife	328 (60.3)	1,511 (54.2)	
Cultivation	75 (13.8)	264 (9.5)	
Business and others	141 (25.9)	1,011 (36.3)	
Smoking, n (%)			0.903
Smoker (current or past)	100 (18.1)	434 (18.4)	
NSAID use, n (%)	7 (1.3)	6 (0.2)	0.002
History of acute gastroenteritis^b^	12 (2.2)	56 (0.2)	<0.001
Presence of psychosocial stress, (cutoff value 23), n (%)	33 (6.0)	28 (1.0)	<0.001
Somatic symptoms (median score, range)	2.0 (0–18)	1.0 (0–15)	<0.001
Anxiety and insomnia (median score, range)	2.0 (0–14)	0.0 (0–16)	<0.001
Social dysfunction (median score, range)	7.0 (0–16)	7.0 (0–15)	0.485
Depression (median score, range)	0.0 (0–13)	0.(0–10)	<0.001
Total score (median score, range)	9.0 (0–45)	8 (0–40)	<0.001

NSAID, nonsteroidal anti-inflammatory drug; UD, uninvestigated dyspepsia.

a 1 US$ = 84.84 taka (dated 30.08.2020).

b Acute gastroenteritis in the past 1 year.

Since the study was conducted in only 3 villages of Bangladesh, we estimated the standardized UD prevalence by adjusting the age—and sex—on the whole population of the country based on the population census of Bangladesh in 2011 (22). The age- and sex-adjusted prevalence rates of UD were 16.2% and 16.1%, respectively. After adjusting for age, the prevalence of UD among men and women was 13.8% and 17.8%, respectively.

### Multivariate analysis of risk factors for UD

On multivariate analysis, age >50 years, female sex, being married, lower family income, nonsteroidal anti-inflammatory drug and aspirin use in the past 3 months, psychological distress, and history of AGE in the past year were found to be the risk factors for UD (Table 2).

**Table 2.** Multivariate analysis of risk factors for UD^a^

Variables	Crude OR (95% CI)	Adjusted OR (95% CI)	*P* value
Age			
Less than 50 yr	Reference	Reference	
50 yr or above	1.29 (1.05–1.6)	1.34 (1.07–1.68)	0.013
Sex			
Male	Reference	Reference	
Female	1.35 (1.11–1.63)	1.42 (1.17–1.74)	<0.001
Marital status			
Single	Reference	Reference	
Married	1.56 (1.21–2.04)	1.57 (1.21–2.07)	<0.001
Family income^b^			
Higher income	Reference	Reference	
Lower income	1.77 (1.43–2.21)	1.79 (1.43–2.26)	<0.001
Use of NSAIDs and aspirin			
Absent	Reference	Reference	
Present	7.05 (2.23–22.3)	7.05 (2.11–23.55)	0.002
Psychological distress			
Absent	Reference	Reference	
Present	6.46 (3.81–10.93)	5.02 (2.87–8.76)	<0.001
Acute gastroenteritis			
Absent	Reference	Reference	
Present	9.3 (3.43–25.25)	5.42 (1.83–16)	0.002
Education			
Up to class V	Reference	Reference	
Class VI and above	0.82 (0.68–0.99)	1.07 (0.87–1.31)	0.546

CI, confidence interval; OR, odds ratio; NSAIDs, nonsteroidal anti-inflammatory drugs; UD, uninvestigated dyspepsia.

a Age, sex, marital status, family income, use of NSAIDs and aspirin, presence of psychological distress, history of acute gastroenteritis, and education were adjusted by stepwise logistic regression to estimate adjusted OR and *P* value.

b Lower family income ,10,000 taka/mo and higher family income .10,000 taka/mo. 1 US$ = 84.84 taka (dated August 30, 2020).

### Comparison of the dyspeptic subjects who did and did not undergo endoscopy

Of 547 subjects, 346 (63.25%) agreed and underwent UGI endoscopy. There were no differences in sociodemographic and clinical characteristics between subjects who did and did not undergo endoscopy except for the fact that subjects with PDS, EPS-PDS overlap, and psychological stress (GHQ-28 score ≥23) agreed to undergo endoscopy more often (Table 3).

**Table 3.** Baseline characteristics of the 547 dyspeptic subjects undergoing and not undergoing endoscopy

Characteristics	Subjects undergoing endoscopy (n = 346)	Subjects not undergoing endoscopy (n = 201)	*P* value
Age, (yr, mean ± SD)	42.65 ± 14.36	43.02 ± 14.56	0.508
Male sex n (%)	138 (39.9)	63 (31.3)	0.053
Marital status, n (%)			0.446
Married	294 (85)	176 (87.6)	
Single	52 (15)	25 (12.4)	
Education, n (%)			0.529
Illiterate and up to class V	198 (57.2)	121 (60.2)	
Class V and above	148 (42.8)	80 (39.8)	
Family income (taka/mo)^a^, mean ± SD	9,790.09 ± 5,587.39	9,851.50 ± 7,016.92	0.908
Occupation, n (%)			0.262
Housewife	200 (58.3)	128 (63.7)	
Cultivation	56 (16.3)	19 (9.5)	
Business and others	87 (25.6)	54 (27.0)	
Smoker (current or past), n (%)	68 (19.8)	32 (16.8)	0.419
Presence of psychosocial stress n (%) (cutoff value 23)	29 (8.4)	4 (2)	0.002
Total GHQ-28 score (mean ± SD)	12.15 ± 7.79	9.39 ± 6.04	0.000
Presence of EPS only, n (%)	53 (15.3)	35 (17.4)	0.547
Presence of PDS only, n (%)	115 (33.2)	86 (42.8)	0.028
Presence of EPS-PDS overlap, n (%)	178 (51.4)	80 (39.8)	0.010

a 1 US$ = 84.84 taka (dated August 30, 2020).

EPS, epigastric pain syndrome; PDS, postprandial distress syndrome.

### Endoscopic findings of dyspeptic patients

**Figure 2.**
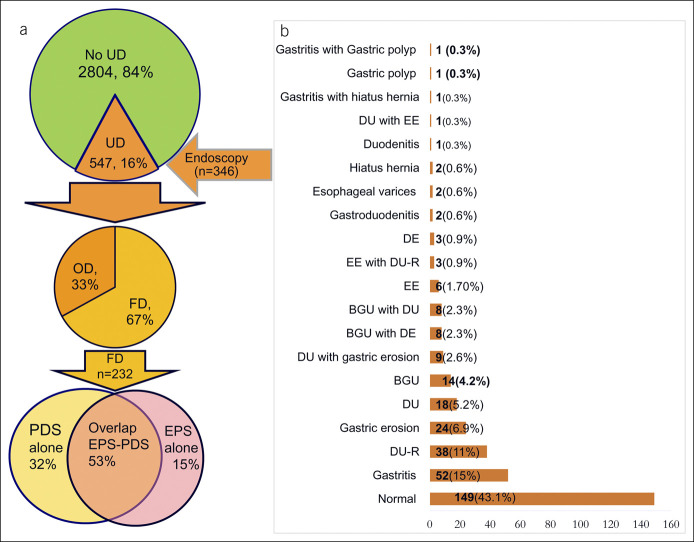
Subtyping of dyspepsia and endoscopic findings of dyspeptic subjects and (**a**) frequency of PDS, EPS, and PDS-EPS overlap (**b**) endoscopic findings of dyspeptic subjects. BGU, benign gastric ulcer; DE, duodenal erosion; DU-R, duodenal ulcer disease in remission; EE, erosive esophagitis; EPS, epigastric pain syndrome; FD, functional dyspepsia; OD, organic dyspepsia; PDS, postprandial distress syndrome; UD, uninvestigated dyspepsia.

At endoscopy, 67% subjects had FD, and 33% had organic lesions (Figure 2a). Figure 2b shows the endoscopic findings. Of 346 dyspeptic subjects, 28.6% had PU (benign gastric ulcer and DU), 3.8% had EE, and 1.2% had PU and EE. Of the 99 subjects with PU (age 45.71 ± 15.17 years), 54 (54.5%) were men; 69 (69.7%) had DU, 22 (22.2%) had benign GU, and 8.1% had both benign GU and DU. Of the PU subjects, 28 (28.28%) had active DU, and 41 (41.41%) had healed DU.

### *H. pylori* infection among dyspeptic subjects

Multiplex PCR, RUT, and histology for *H. pylori* in gastric biopsies were performed in 268, 342, and 342 subjects, respectively. *H. pylori* were positive among 266 (78%) of the 342 dyspeptic subjects undergoing endoscopy.

### Comparisons of the subjects with FD and OD

Table 4 shows the comparisons of FD and OD subjects. FD was more common among females and homemakers, whereas OD was more common among men, cultivators, and other professions. The use of nonsteroidal anti-inflammatory drugs and aspirin was more common in OD than FD subjects. The frequency of EPS, PDS, EPS-PDS overlap, psychological stress, and *H. pylori* infection was comparable between FD and OD. However, the median GHQ-28 score was higher among FD (11.0) compared with OD subjects (9.0) (*P* = 0.021). The median anxiety and insomnia scores were also higher among FD (2.0) compared with OD subjects (0.0) (*P* ≤ 0.001).

**Table 4.** Sociodemographic, clinical, and psychological characteristics and QoL issues of subjects with FD and OD

Characteristics	FD (total = 232), n (%)	OD (total = 114), n (%)	*P* value
Age			0.104
Less than 50 yr	174 (75)	76 (66.7)	
Age 50 yr or above	58 (25)	38 (33.3)	
Sex			<0.001
Male	76 (32.8)	62 (54.4)	
Female	156 (67.2)	52 (45.6)	
Marital status			0.752
Married	196 (84.5)	98 (86)	
Single	36 (15.5)	16 (14)	
Education, n (%)			0.133
Illiterate and up to class V	126 (54.3)	72 (63.2)	
Class VI and above	106 (45.7)	42 (36.8)	
Family income^a^			0.732
Lower income	178 (76.7)	87 (78.4)	
Higher income	54 (23.3)	24 (21.6)	
Occupation			0.002
Homemaking	152 (65.5)	49 (43.0)	
Cultivation	28 (12.1)	29 (24.3)	
Business and others	52 (22.4)	36 (31.6)	
Religion			0.386
Muslim	157 (68)	83 (72.8)	
Hindu	74 (32)	31 (27.2)	
History of smoking (current or past)	38 (16.5)	30 (26.5)	0.031
History of acute gastroenteritis	54 (23.3)	20 (17.5)	0.222
Use of NSAIDs and aspirin	3 (1.3)	8 (7)	0.004
Dyspepsia subtype			0.538
EPS alone	35 (15.1)	18 (15.8)	
PDS alone	73 (31.5)	42 (36.8)	
EPS-PDS overlap	124 (53.4)	54 (47.4)	
*H. pylori* positivity	174 (75)	92 (80.7)	0.174
IBS	66 (28.4)	26 (22.8)	0.264
Heartburn	88 (37.9)	44 (38.6)	0.905
Presence of psychological distress	22 (9.5)	7 (6.1)	0.292
Impaired QoL			0.906
Abdominal pain	87 (57.5)	44 (38.6)	0.480
Meal-related symptoms	55 (23.7)	28 (24.6)	

EPS, epigastric pain syndrome; FD, functional dyspepsia; IBS, irritable bowel syndrome; QoL, quality of life; NSAIDs, nonsteroidal anti-inflammatory drugs; OD, organic dyspepsia; PDS, postprandial distress syndrome.

a Lower family income, 10,000 taka/mo and higher family income. 10,000 taka/mo.1 US$ = 84.84 taka (dated August 30, 2020).

### Subtyping of dyspeptic subjects into EPS, PDS, and EPS-PDS overlap

Of 547 UD participants, 201 (37%), 88 (16%), and 258 (47%) had PDS alone, EPS alone, and PDS-EPS overlap, respectively. The prevalence of PDS alone, EPS alone, and EPS-PDS overlap was 6%, 2.6%, and 7.7%, respectively. About 53% of FD subjects could not be categorized either into EPS or PDS, 32% had PDS, and only 15% had EPS (Figure 2a). Table 5 shows the characteristics of FD subjects with EPS, PDS, and EPS-PDS overlap. There were no differences in sociodemographic characteristics and frequency of *H. pylori* infection among the FD subjects with EPS alone, PDS alone, and EPS-PDS overlap. The number of subjects with psychological stress was comparable among the 3 groups of FD subjects. However, the total GHQ-28 and social dysfunction scores were higher among subjects with EPS compared with PDS and EPS-PDS overlap.

**Table 5.** Sociodemographic and psychological characteristics of FD subjects with EPS, PDS, and EPS-PDS overlap

Characteristics	EPS only (n = 35)	PDS only (n = 73)	PDS-EPS overlap (n = 124)	*P* value
Age, (yr, mean ± SD)	38.11 ± 15.50	43.33 ± 15.05	40.57 ± 12.67	0.615
Male sex n (%)	11 (31.4)	26 (35.6)	39 (31.5)	0.821
Marital status, n (%)				0.577
Married	31 (88.6)	63 (86.3)	102 (82.3)	
Single	4 (11.4)	10 (13.7)	22 (17.7)	
Education, n (%)				0.289
Illiterate and up to class V	15 (42.9)	43 (58.9)	68 (54.8)	
Class V and above	20 (57.1)	30 (41.1)	56 (45.2)	
Family income (taka/mo)^a^, mean ± SD	10,857.14 ± 6,283.23	9,904.11 ± 5,826.58	10,056.45 ± 5,836.95	0.954
Occupation, n (%)				0.258
Housewife	23 (65.7)	44 (62.0)	84 (67.7)	
Cultivation	5 (14.3)	11 (15.5)	12 (9.7)	
Business and others	7 (20.0)	16 (22.5)	28 (22.6)	
Religion, n (%)				0.719
Muslim	22 (62.9)	49 (67.1)	157 (68)	
Hindu	13 (37.1)	24 (32.9)	74 (32)	
Smoking (current or past), n (%)	7 (20.0)	12 (16.9)	19 (15.3)	0.801
*H. pylori* positivity	29 (82.9)	57 (78.1)	88 (71.0)	0.273
Presence of psychological stress, n (%) (cutoff value 23)	4 (11.4)	7 (9.6)	11 (8.9)	0.901
Somatic symptoms (median score)	4.0 (0–15)	3.0 (0–14)	3.0 (0–18)	0.241
Anxiety and insomnia (median score, range)	1.0 (0–14)	3.0 (0–14)	2.0 (0–13)	0.145
Social dysfunction (median score, range)	7.0 (3–11)	7.0 (0–14)	7.0 (0–16)	0.03
Depression (median score, range)	0.0 (0–5)	0.0 (0–8)	0 (0–13)	0.04
Total score (median score, range)	12.0 (6–40)	12.0 (0–43)	9.5 (0–45)	0.048

a 1 US$ = 84.84 taka (dated August 30, 2020).

EPS, epigastric pain syndrome; FD, functional dyspepsia; PDS, postprandial distress syndrome.

### Virulence factors of *H. pylori* among subjects with FD and PU

Of 268 dyspeptic individuals undergoing multiplex PCR for *H. pylori*, it was detected among 244 (91.04%) subjects. *H. pylori*–infected patients with PU had a higher frequency of CagA and vac genotype s1m1 positivity than those with FD (*P* < 0.05), as shown in Supplementary Table 1, Supplementary Digital Content 1, http://links.lww.com/CTG/A568.

### Frequency of FH and IBS among patients with UD

**Figure 3.**
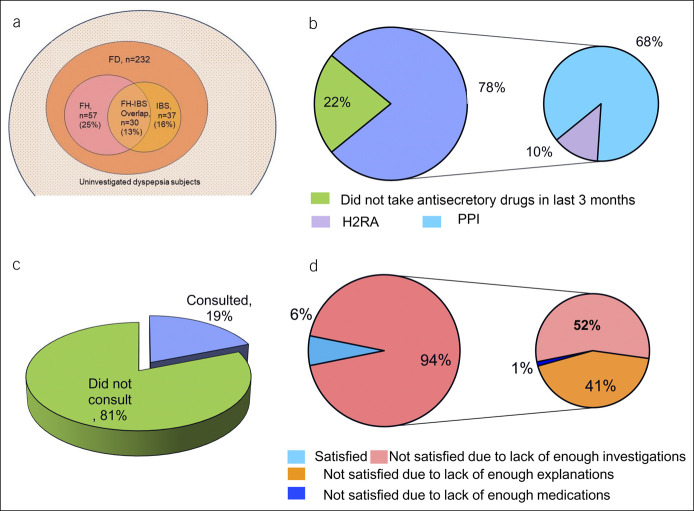
Consultation rate, medication use, satisfaction with treatment, and overlap of UD with FH and IBS. (**a**) Frequency of FH and IBS among subjects with FD. (**b**) Medication use, (**c**) consultation rate, and (**d**) satisfaction with treatment. FD, functional dyspepsia; FH, functional heartburn; H2RA, histamine receptor 2 antagonists; IBS, irritable bowel syndrome; PPI, proton pump inhibitor; UD, uninvestigated dyspepsia.

Of the 547 UD subjects, 203 (37%) had FH, 110 (20%) had IBS, and 52 (9.5%) had both heartburn and IBS. The prevalence of FH-UD overlap, UD-IBS overlap, and FH-UD-IBS overlap was 6.1%, 3.3%, and 1.6%, respectively. Among FD subjects, 54% had overlap with either FH or IBS or both; 25% had overlap with FH only, 13% both FH and IBS, and 16% had overlap with IBS only as shown in Figure 3a.

### QoL issues

Abdominal pain, early satiety, and abdominal fullness affected QoL significantly (a lot and a great deal) in about one-third of the UD subjects, as shown in Supplementary Figure 1, Supplementary Digital Content 2, http://links.lww.com/CTG/A567. Abdominal discomfort or bloating associated with dyspeptic symptoms affected the QoL in 42.1% and 40.5% dyspeptic subjects, respectively. Abdominal pain and meal-related symptoms affected the QoL among FD and OD subjects similarly (Table 4).

### Medication use, consultations, and satisfaction with treatment

Of the dyspeptic subjects, 428 (78.4%) took antisecretory drugs during the past 3 months; 372 (68.1%) and 56 (10.3%) took proton pump inhibitors and H_2_ receptor antagonist, respectively (Figure 3b). Among the subjects with FD and OD, 179 (77.2%) and 86 (75.4%) took either proton pump inhibitors or H_2_ receptor antagonist, respectively, in the past 3 months (*P* = 0.723). Of the subjects with UD, 104 (19.0%) consulted healthcare professionals with comparable frequency among the male and female subjects (35/201, 17.4% vs 69/346 19. %; *P* = 0.49) (Figure 3c). Of the 538 UD subjects, only 6.3% were satisfied with their treatment. The reasons for dissatisfaction were lack of enough investigations and lack of enough explanation in most of the dyspeptic subjects and not having enough medications in the minority (Figure 3d). Among the subjects with FD and OD, 15 (6.5%) and 7 (6.2%) were satisfied with their treatment, respectively (*P* = 0.88).

## DISCUSSION

This cross-sectional study conducted in a Bangladeshi Asian rural community demonstrates that about 16% of the population had UD by Rome III criteria, half of them had EPS-PDS overlap, and EPS alone was uncommon; about one-third of the UD subjects had OD (PU being the most frequent cause and EE was uncommon). About half of the FD subjects could not be categorized into either EPS or PDS. There was no difference in sociodemographic characteristics, psychological stress, and *H. pylori* infection among the FD patients with EPS, PDS, and EPS-PDS overlap.

A recent global study by the Rome Foundation on the worldwide prevalence and burden of FGIDs found the prevalence of UD to be 7.2% by internet survey and 4.8% by household survey using the Rome IV criteria. In this study, the prevalence of UD in Bangladesh was 19.4% (23). This higher prevalence of UD compared with this study may result from the inclusion of the urban population in the global study. In a community-based study in the eastern part of Bangladesh, the prevalence of UD was 11.8% (24). A community-based study in a rural population in India found the prevalence of UD to be 15% (25), which is higher than another study among adolescents in Delhi, India (26). The estimated prevalence of UD in the Western and Eastern population based-on the Rome III criteria was 9.8%–20.2% and 5.3%–12.8%, respectively, in a recent review (5). The global prevalence of UD was 21% (95% CI 17.8%–23.9%) in a recent meta-analysis. However, the prevalence varied from 1.8% to 57.0% depending on the definition used and the geographical location (27). This wide variation in prevalence might be related to variations in diagnostic criteria, study population, survey method, survey instrument, cultural and regional differences in symptom interpretation, and reporting.

Globally, there are only a handful of community-based studies on the prevalence of OD and FD. In a community-based study in 2 villages in Italy, 15% and 11% had UD and FD. Among the UD patients, 27% had OD (8). In another community-based endoscopy assisted survey from 2 northern Swedish municipalities, 20% had UD, and 15.7% had FD based on the Rome III criteria (28). The hospital or clinic-based studies in Asia among patients with dyspepsia showed a higher rate of organic lesions at endoscopy (29,30). In a meta-analysis, the prevalence of clinically significant endoscopic findings in individuals with dyspepsia was 27.5% (EE 20% and PU 6%) and 18% (EE 6% and PU 11%) according to broad definitions and Rome I and II criteria (11). The high frequency of organic lesions among UD in this study might be related to the high prevalence of *H. pylori* infection in the Bangladeshi community (31,32).

If we extrapolate that 29% of UD subjects have PU, the prevalence of PU in the study population is estimated to be 5%. A household survey conducted 4 decades ago in Bangladesh using a structured questionnaire found an overall PU prevalence of 16% (33). The lower prevalence of PU in the current study might be related to a reduction in *H. pylori* infection, greater treatment availability, and different UD definitions used. The high prevalence of PU and low prevalence of EE among dyspeptics in this study are consistent with the findings of the meta-analysis that showed that the pooled prevalence of PU was higher (11.0%; 95% CI, 6.0%–19.0%) and EE was lower (2.7%; 95% CI, 1.2%–4.8%) in Asian studies compared with Western studies (6.0%; 95% CI, 5.0%–8.0%) and (25.0%; 95% CI, 4.0%–57.0%), respectively (11). A recent consensus from India reported the frequency of GERD in Indian community studies to be about 7% (34). A prospective study demonstrated that GERD is more common in British compared with South-East Asian dyspeptic patients (35). These differences may result from differences in epidemiology and risk factors for dyspepsia and GERD between Asian and Western patients (5,20).

About half of our study participants with UD or FD cannot be categorized into 2 distinct EPS and PDS types by the current classification. Although a few population-based studies showed a lower frequency of PDS-EPS overlap symptoms than by chance, all these were conducted among the Western population (8,9,36,37). Clinic-based studies from different countries found the more unsatisfactory performance of subtyping FD into EPS and PDS (2,38–40). In the Indian community-based study, among UD subjects, 9% had EPS, 27% PDS, and 64% had EPS-PDS overlap (25). The subtyping of FD patients into PDS and EPS was not validated in the community-based endoscopy assisted to study in Asian population previously (5,41).

The findings of this study show that about one-third of the dyspeptic subjects defined by the Rome criteria had OD; this may suggest that the Rome criteria may not entirely excludes OD in our population. There may be at least 2 reasons for such poor performance. First, the Rome criteria were initially developed and validated mostly in the English-speaking Western population. Symptoms of FGIDs may be influenced by psychosocial and sociocultural factors that affect the illness beliefs, perception, and symptoms reporting (42). Hence, symptoms-based criteria such as the Rome criteria may not do well in non-Western populations. To overcome such shortcoming, a culturally adapted version of the Rome III diagnostic questionnaire developed by Asian experts known as EAR3Q (43), was used in this study. However, such adaptation still may not do well in our population. Second, the Rome criteria probably cannot reliably discriminate between FD and uncomplicated PU. Therefore, the high prevalence of *H. pylori* and PU in the study population might result in poor performance of the Rome criteria.

The high prevalence of OD among dyspeptics in the community in this study and other hospital-based studies in Asia suggests that routine UGI endoscopy may be preferred to empirical treatment or test-and-treat strategy for Asian patients, particularly in low-middle-income countries such as Bangladesh. Moreover, the high prevalence of *H. pylori*, availability of endoscopy at a lower cost, and indifferent sociodemographic and clinical characteristics of FD and OD subjects further support the scope-and-treat as the initial strategy.

This study is perhaps the first population-based endoscopy-assisted community survey in South Asia providing prevalence of FD. Moreover, *H. pylori*–associated virulence factors have been tested in the community. One of the limitations was the use of Rome III than currently proposed Rome IV criteria. The reason was that the study began about 4 years before the Rome IV criteria were released in 2016. Another limitation might be that the study was conducted only in 3 villages of Bangladesh, which might not be representative of the whole country. To overcome such limitations, we standardized the UD prevalence by adjusting the age—and sex—of the whole population of the country based on the population census of Bangladesh in 2011 (22). However, there were no significant differences in the adjusted and crude prevalence of UD, suggesting that the study population may well-represent the Bangladeshi population. The response rate of this community-based study was very high (94.15%). Since, the response rate to participate was quite high, the possibility of response bias, if any, is less likely. Although the community participation was very high and nonresponse was low, we could not provide a comparison of the responder and nonresponder, which might be a limitation. Endoscopy was not performed among all the dyspeptic participants; this might be another limitation. Since endoscopy is an invasive procedure, it is not unexpected that all the dyspeptics in the community may not undergo endoscopy. However, there were no significant differences in sociodemographic and clinical characteristics between subjects who did and did not undergo endoscopy.

In conclusion, 16%, 11%, and 5% of rural adults in Bangladesh had UD, FD, and PU, respectively; half of dyspeptics had PDS-EPS overlap. A third of the subjects with UD had OD, mostly PU. About 29% UD subjects had overlap with IBS and 37% with FH. About 80% of dyspeptic subjects were *H. pylori*–positive. There were no differences in the frequency of *H. pylori* infection between subjects with FD and PU. Subjects with PU had more virulent *H. pylori* compared with those with FD.

## CONFLICTS OF INTEREST

**Guarantor of the article:** M. Masudur Rahman, FCPS, MD, FACP, FACG, FRCP, and Uday C. Ghoshal, MD, DNB, DM, FACG, RFF, FAMS, FRCP.

**Specific author contributions:** M.M.R.: study conceptualization, planning, supervision of conduct, analysis and interpretation of data, drafting of manuscript, and approval of the final manuscript. U.C.G.: study conceptualization and planning, analysis and interpretation of data, drafting of manuscript, and approval of the final manuscript. M.G.K: study planning, conduct of the study, and approval of final manuscript. N.S.: study conceptualization, data collection, and approval of the final manuscript. A.Y.: study planning, conduct of the study, and approval of final manuscript. S.N: study planning, conduct of the study, drafting of manuscript, and approval of final manuscript. F.A.: study planning, conduct of the study, and approval of the final manuscript. AHM.R.: study planning, supervision of conduct, and approval of the final manuscript. M.H.: study conceptualization, planning, conduct of the study, editing of the manuscript, and approval of the final manuscript.

**Financial support:** Bangladesh Medical Research Council (BMRC/HPNSDP/research grant/2011 -2012/474).

**Conflicts of interest:** None to report.

**Study Highlights**

**WHAT IS KNOWN**

✓ Uninvestigated dyspepsia is common in the community.
✓ The true prevalence of functional dyspepsia is rarely reported.

**WHAT IS NEW HERE**

✓ About one-third of the uninvestigated dyspeptic subjects has organic dyspepsia (OD) in a rural community in South Asia.
✓ Most participants with OD had peptic ulcer.
✓ About half of the participants with functional dyspepsia could not be categorized into epigastric pain syndromes or postprandial distress syndrome.

**TRANSLATIONAL IMPACT**

✓ High frequency of OD in the community suggests that routine upper gastrointestinal endoscopy may be preferred to empirical treatment with proton pump inhibitor or test-and-treat in some Asian countries.

## Supplementary Material

SUPPLEMENTARY MATERIAL
