# Supplementary material for: Functional Dyspepsia, Peptic Ulcer, and Helicobacter pylori Infection in a Rural Community of South Asia: An Endoscopy-Assisted Household Survey
Source: Clin Transl Gastroenterol. 2021 Apr 16;12(4):e00334. doi: 10.14309/ctg.0000000000000334 (PMC8052092; doi:10.14309/ctg.0000000000000334)
Supplement: SUPPLEMENTARY MATERIAL [file ct9-12-e00334-s001.pdf]

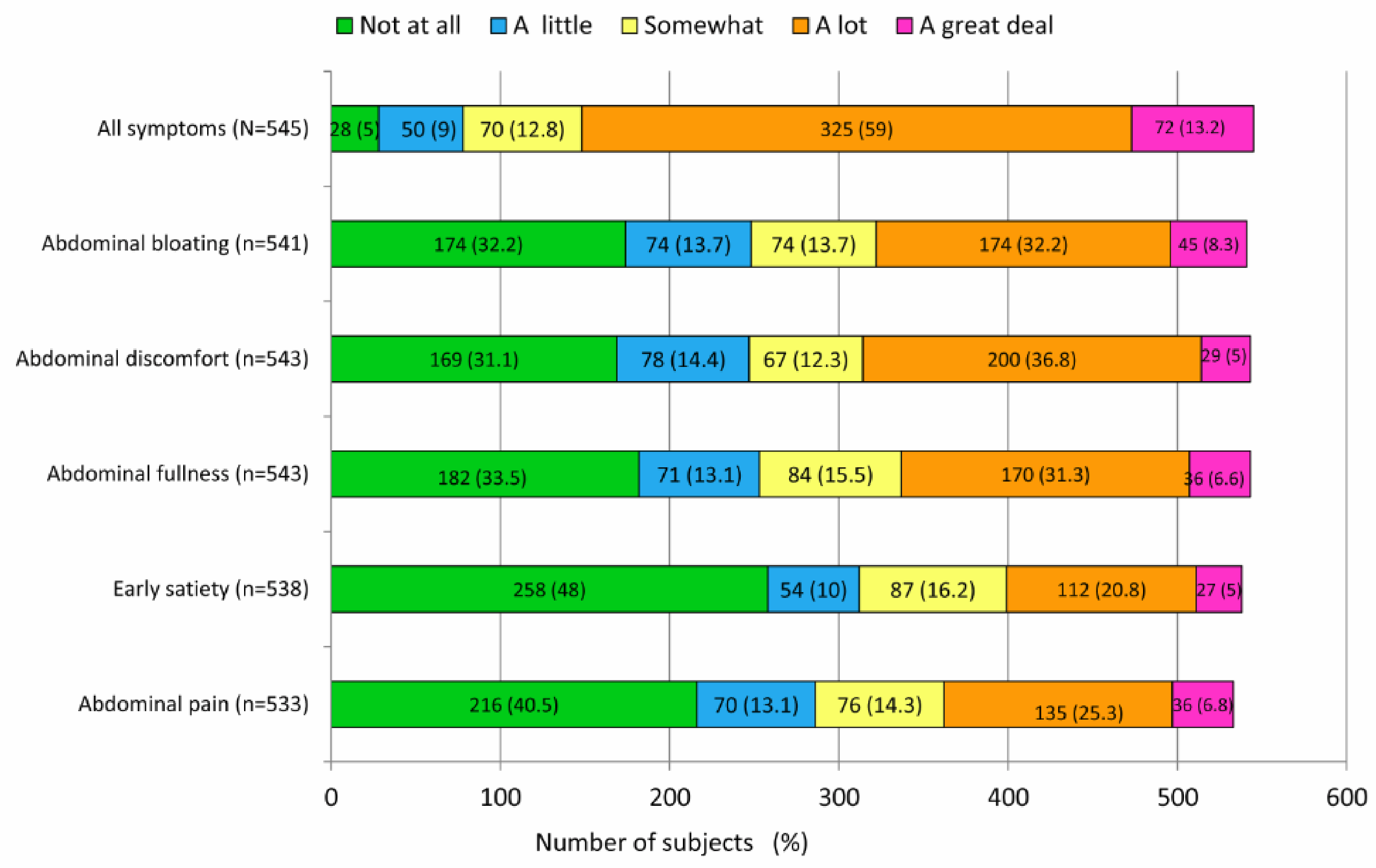

Figure Supplemental Digital Content 2: Symptoms affecting the quality of life (QoL) among the subjects with uninvestigated dyspepsia.
