# Supplementary material for: Functional Dyspepsia, Peptic Ulcer, and Helicobacter pylori Infection in a Rural Community of South Asia: An Endoscopy-Assisted Household Survey
Source: Clin Transl Gastroenterol. 2021 Apr 16;12(4):e00334. doi: 10.14309/ctg.0000000000000334 (PMC8052092; doi:10.14309/ctg.0000000000000334)
Supplement: SUPPLEMENTARY MATERIAL [file ct9-12-e00334-s002.docx]

**Supplementary Table 1: Cag A and Vac A positivity among patients with functional dyspepsia (FD) and peptic ulcer**

| **Virulence factors** | **Functional dyspepsia**  **(total 170)**  **% (N)** | **Peptic ulcer**  **(total 74)**  **% (N)** | ***P*- value** | **All dyspeptic patients (FD and PU- total 244)**  **% (N)** |
| --- | --- | --- | --- | --- |
| CagA +ve | 43.5% (74) | 70.36 % (52) | 0.000 | 51.6% (126) |
| Vac A s1m1* | 38.8% (66) | 64.9% (48) | 0.000 | 46.72% (114) |
| Vac A s1m2** | 28.2% (48) | 20.3% (15) | 0.207 | 25.81% (63) |
| Vac A s2m1** | 3.5% (6) | 1.4% (1) | 0.678 | 2.86% (7) |
| Vac A s2m2*** | 4.1% (7) | 1.4% (1) | 0.441 | 3.28% (8) |

- **Most virulent,** ****intermediate virulent,** *****non-virulent**
